# Current status and capacity of pathogen laboratories in centers for disease control and prevention in China during the COVID-19 pandemic: A nationwide cross-sectional survey

**DOI:** 10.3389/fpubh.2022.927318

**Published:** 2022-08-11

**Authors:** Ji Wang, Peihua Niu, Ruiqing Zhang, Jingyi Li, Mingzhu Nie, Xuejun Ma

**Affiliations:** ^1^National Institute for Viral Disease Control and Prevention, Chinese Center for Disease Control and Prevention, Beijing, China; ^2^Chinese Field Epidemiology Training Program, Chinese Center for Disease Control and Prevention, Beijing, China; ^3^Joint Research Centre for Emerging Infectious Diseases and Biosecurity, Chinese Academy of Sciences, Wuhan, China

**Keywords:** capacity, pathogen laboratory, centers for disease control and prevention, COVID-19, cross-sectional survey

## Abstract

The pathogen laboratory (p-lab) is the core and primary department of centers for disease control and prevention (CDCs) in China to respond to infectious disease outbreaks such as COVID-19. To understand the current status and capacity of p-labs in Chinese CDCs during the COVID-19 pandemic, we conducted a nationwide cross-sectional survey among 399 respondents from 239 CDCs. Differences in the current status of p-labs in CDCs of provinces, cities, and counties mainly comprised laboratory equipment, IEIs, mastery of personal occupational skills, and maximum detection capacity. Most CDCs reported a lack of staff and funds for personnel, which should be a priority in China's upcoming public health reform. The development of sequencing technologies has received considerable attention in CDCs. These are mainly used to study respiratory viruses such as influenza and SARS-CoV-2. The COVID-19 pandemic has driven development of the CDCs in China, and personnel and funds are considered key factors in improving the detection capacity of CDC p-labs.

## Introduction

Laboratory services are an essential and fundamental part of public health systems. With the occurrence of infectious disease outbreaks such as the COVID-19 pandemic or other public health events, centers for disease control and prevention (CDCs) are at the heart of public health investigation and response mechanisms (1). According to data released by the National Bureau of Statistics in 2020, there are 34 provincial-level, 333 city-level, and 2,844 county-level administrative divisions in China, each of which usually has an independent CDC providing public health services for the corresponding jurisdiction.

The pathogen laboratory (p-lab) is the core and primary department of CDCs in the response to infectious disease outbreaks. The capacity of p-labs is a comprehensive manifestation of various aspects including a functional organization structure, appropriate testing services, infrastructure, human resources, reagent and equipment procurement, and supply systems. The International Health Regulations (IHR, 2005)^1^ have placed specific responsibilities on World Health Organization (WHO) Member States to build and strengthen their capacities in confronting all potential public health emergencies of international concern. Thus, p-labs have a critical role in this surveillance and response process (2, 3). Within this framework, it is necessary to use a standardized approach and methodology to investigate and evaluate the capacity of p-labs within China's CDCs (4, 5).

The objective of this study was to investigate the current status and evaluate the CDC p-lab capacity in China via a nationwide cross-sectional survey to compare the changes before and after the COVID-19 pandemic, to preliminarily explore the factors affecting p-lab capacity and discuss the possible direction of development of CDC p-labs in the future.

## Methods

### Respondents and survey

Survey respondents were laboratory staff from China's provincial, city, and county CDCs who engaged in pathogen detection-related work. Approximately 10 respondents were selected from each province using stratified sampling. Approximately 20 respondents from county-level CDCs were randomly selected in two representative provinces (Jiangxi and Hainan, which represent moderately developed and underdeveloped provinces, respectively). An electronic questionnaire including 14 single-choice questions, four multiple-choice questions, and four open-ended questions was designed based on WHO guidelines (4) and administered on line to respondents via a WeChat application in June 2021. The time to complete the questionnaire was limited to ~1 week.

### Metrics for evaluation

Laboratory equipment penetration (LEP) was defined as the proportion of CDCs possessing a certain kind of laboratory equipment (e.g., −70°C freezer) among the total CDCs investigated. The implementable rate (IR) was defined as the proportion of CDCs that could complete a certain kind of implementable experimental item (IEI; e.g., vaccine development) among the total CDCs investigated. The implementable experimental item score (IEI-S) was defined as the number of types of IEI that a CDC could complete (0–12). Mastery was defined as the proportion of respondents who mastered a certain kind of personal occupational skill (POS; e.g., primer design) among all respondents surveyed. The personal occupational skill score (POS-S) was defined as the number of types of POS that a respondent had mastered (0–12). Maximum detection capacity (MDC) was defined as the maximum number of swab samples that can be detected daily using quantitative real-time polymerase chain reaction (q-PCR) at a CDC.

### Statistical methods

The geographical coordinates of the CDC to which each respondent was affiliated were acquired from Baidu Maps (the Chinese equivalent of Google Maps), and the map was created using ArcGIS. The funds for reagents, equipment, and staff were rated by respondents using a five-level Likert scale (totally insufficient, relatively insufficient, just enough, relatively sufficient, very sufficient). A *P* < 0.05 was considered statistically significant in this study. MDC was regarded as an interval variable for descriptive statistics, and the midpoint of each range was taken as the approximation of the actual MDC (e.g., 1,500 was taken for the interval 1,000–2,000) for non-parametric and correlation tests. Normality was assessed using the Shapiro–Wilk test. The Kolmogorov–Smirnov test or Kruskal–Wallis test was performed to determine statistically significant differences between groups. Correlations were assessed using the Spearman coefficient. A word cloud visually depicting the word frequency in the answers to open-ended questions was generated online (weiciyun.com), and the top 15 words were expressed via histogram.

## Results

### Survey respondents

A total of 410 questionnaires were completed, of which 399 were valid, giving an effective rate of 97.3%. The 399 respondents were from 239 different CDCs in China and were engaged in pathogen detection-associated work.

**Figure 1:**
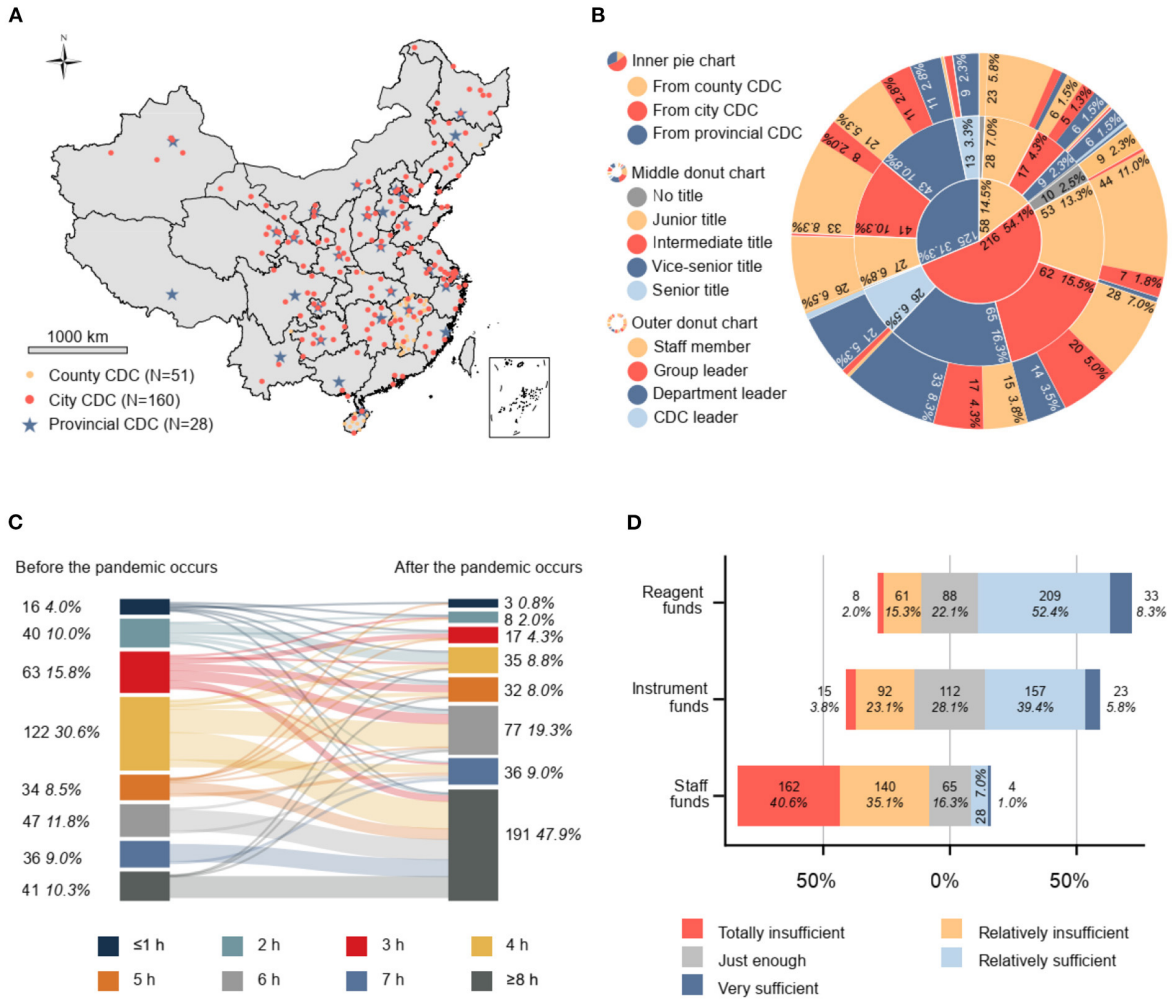
**(A)** Distribution of sampling sites in China (*N* = 239). **(B)** Characteristics of the study population (*N* = 399). **(C)** Number and proportion of p-lab staff with average working duration before and after the COVID-19 outbreak (*N* = 399). **(D)** Divergent stacked bar chart visualizing five-level Likert scale for evaluation of funds; 1: totally insufficient, 2: relatively insufficient, 3: just enough, 4: relatively sufficient, 5: very sufficient. The bar graph extends from the neutral position (gray) toward both ends representing ample (blue and dark blue) and inadequate (yellow and red). The length of the color represents the proportion of respondents who chose this response among all 339 respondents; the starting point of each bar chart is different, with a total length of 100%. CDC, center for disease control and prevention.

Of the 239 CDCs surveyed in this study, 11.7% (28/239) were provincial-level CDCs, 66.9% (160/239) were city-level CDCs and 21.3% (51/239) were county-level CDCs, accounting for 82.4% (28/34), 48.0% (160/333), and 1.8% (51/2,844) of the corresponding level of CDC throughout China. As shown in Figure 1A, the provincial and city CDCs surveyed were dispersed throughout various districts whereas the county-level CDCs were mainly from two provinces (Jiangxi and Hainan) with moderate public health competency in China, making the results nationally representative.

Among all 399 respondents surveyed (Figure 1B), 31.3% (125/399), 54.1% (216/399), and 14.5% (58/399) were p-lab staff from provincial-, city-, and county-level CDCs, respectively. A total 3.5% (14/399) of respondents had no professional title, 27.1% (108/399) had a junior title, 30.1% (120/399) had an intermediate title, 29.3% (117/399) had a vice-senior title, and 10.0% (40/399) of respondents had a senior title. The proportion of ordinary laboratory staff, team leaders, department leaders, and CDC leaders among the total respondents was 53.1% (212/399), 19.8% (79/399), 26.3% (105/399), and 0.8% (3/399), respectively.

### Current status of p-labs

#### Organization and management

The average working duration of p-lab staff has increased by at least 2 h (4.5–6.5 h) per day to deal with the abrupt increase in the SARS-CoV-2 testing-related workload. Most respondents worked 4 h (30.6%, 122/399), 3 h (15.8%, 63/399), and 6 h (11.8%, 47/399) per day before the COVID-19 outbreak, which increased to ≥8 h (47.9%, 191/399), 6 h (19.3%, 77/399), and 7 h (9.0%, 36/399) in the context of the COVID-19 pandemic (Figure 1C).

The available working funds for SARS-CoV-2 testing reagents and consumables (reagent funds) were considered sufficient by 60.7% (242/399), just enough by 22.1% (88/399), and insufficient by 17.3% (69/399) of respondents. Funds for p-lab equipment purchasing and maintenance (equipment funds) were considered sufficient by 45.1% (180/399), just enough by 28.1% (112/399), and insufficient by 26.8% (107/399) of respondents. Funds for personnel expenditure covering salaries, work allowances, staff benefits, and overtime pay (staff funds) were considered sufficient by 8.0% (32/399), just enough by 16.3% (65/399), and insufficient by 75.7% (302/399) of respondents. A considerable proportion of respondents (40.6%, 162/399) indicated that their CDC was severely deficient in terms of staff funds. See Figure 1D for further details.

#### Laboratory equipment penetration (LEP)

**Figure 2:**
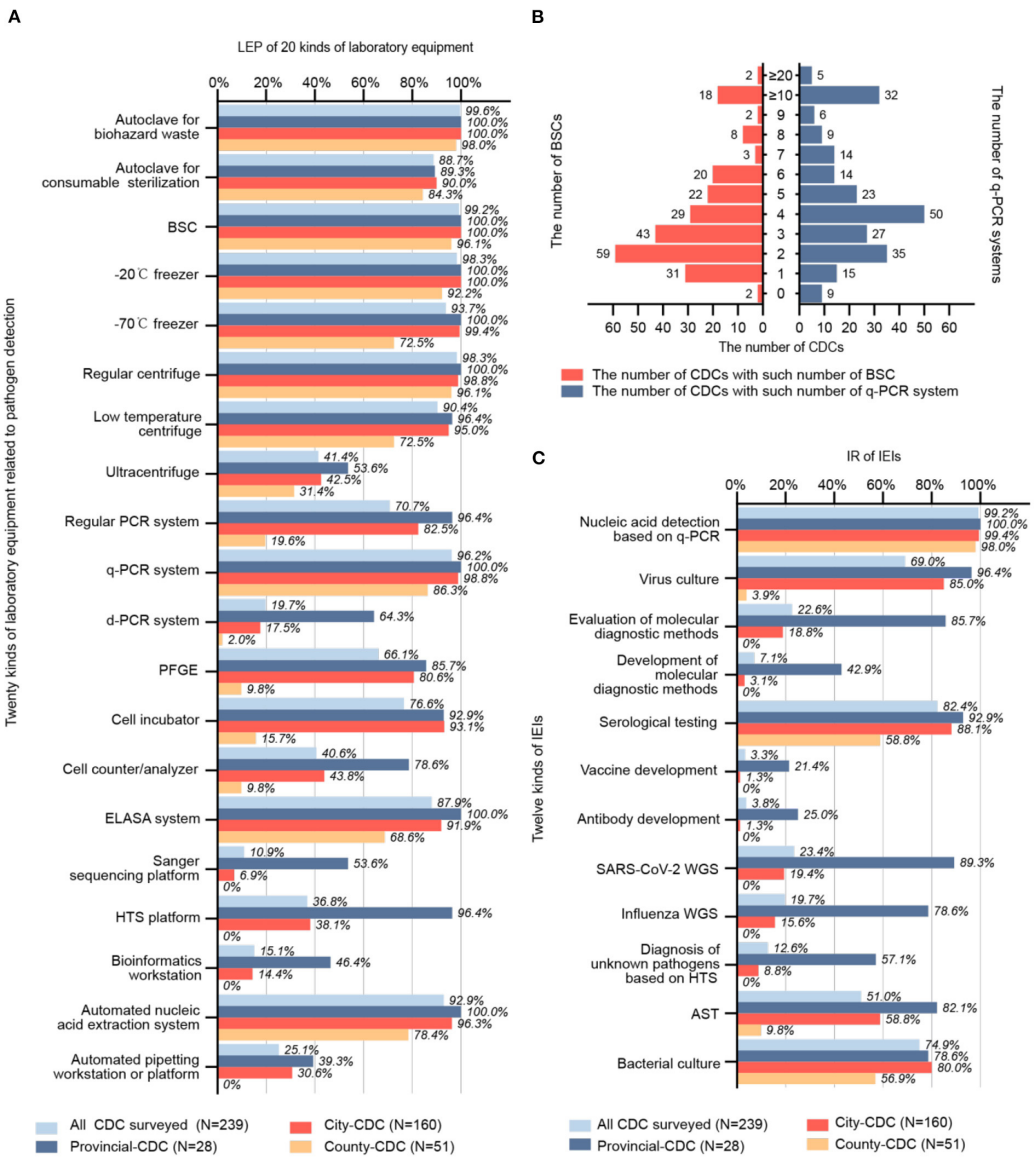
**(A)** LEP of 20 different kinds of laboratory equipment (*N* = 239). **(B)** Number and allocation of BSC and q-PCR systems (*N* = 239). **(C)** IR of 12 kinds IEI (*N* = 239). LEP, laboratory equipment penetration; CDC, center for disease control and prevention; BSC, biosafety cabinet; q-PCR; quantitative real-time polymerase chain reaction; IR, implementable rate; IEI, implementable experimental item; AST, antimicrobial susceptibility test; WGS, whole genome sequencing; HTS, high-throughput sequencing; ELISA, enzyme-linked immunosorbent assay; PFGE, pulsed-field gel electrophoresis.

We investigated the LEP for 20 kinds of lab equipment (Figure 2A), which can be classified into six major categories: biosafety, centrifugation, nucleic acid detection, immunology, sequencing, and laboratory automation. Equipment with a high LEP (>80%) included autoclave for biohazard waste (99.6%), autoclave for consumables sterilization (88.7%), biosafety cabinet (BSC; 99.2%), −20°C freezer (98.3%), −70°C freezer (93.7%), regular centrifuge (98.3%), low-temperature centrifuge (90.4%), q-PCR system (96.2%), enzyme-linked immunosorbent assay (ELISA) system (87.9%), and automated nucleic acid extraction system (92.9%). Equipment with a relatively high LEP (60–80%) included regular PCR system (70.7%), pulsed-field gel electrophoresis (PFGE, 66.1%), and cell culture incubator (76.6%). Equipment with an intermediate LEP (40–60%) included ultracentrifuge (41.4%) and cell counter/analyzer (40.6%). Equipment with a relatively low LEP (20–40%) included high-throughput sequencing platform (HTS platform; 36.8%) and automated pipetting workstation or platform (25.1%). Equipment with a low LEP (<20%) included a digital PCR system (d-PCR, 19.7%), Sanger sequencing platform (10.9%), and bioinformatics workstation (15.1%).

The mode of the number of BSCs per CDC was 2, with a median of 3, Q1 of 2, Q3 of 5, and P90 of 8. The mode of the number of q-PCR systems per CDC was 4, with a median of 4, Q1 of 3, and Q3 of 7; the P90 was unavailable to locate within the interval of ≥10. Having two BSCs and four q-PCR systems was most common for a CDC. Details are shown in Figure 2B.

#### Implementable rate (IR)

The IR of 12 different IEIs was investigated (Figure 2C). IEIs with a high IR (>80%) included nucleic acid detection based on q-PCR (99.2%) and serological testing (82.4%). IEIs with a relatively high IR (60–80%) included virus culture (69.0%) and bacterial culture (74.9%). The only IEI with an intermediate IR (40–60%) was antimicrobial susceptibility test (AST, 51.0%). IEIs with a relatively low IR (20–40%) included evaluation of molecular diagnostic methods (22.6%) and SARS-CoV-2 whole genome sequencing (WGS, 23.4%). IEIs with a low IR (<20%) included development of molecular diagnostic methods (7.1%), vaccine development (3.3%), antibody development (3.8%), WGS of influenza viruses (19.7%), and diagnosis of unknown pathogens based on HTS (12.6%).

#### Training and practice for p-lab staff

**Figure 3:**
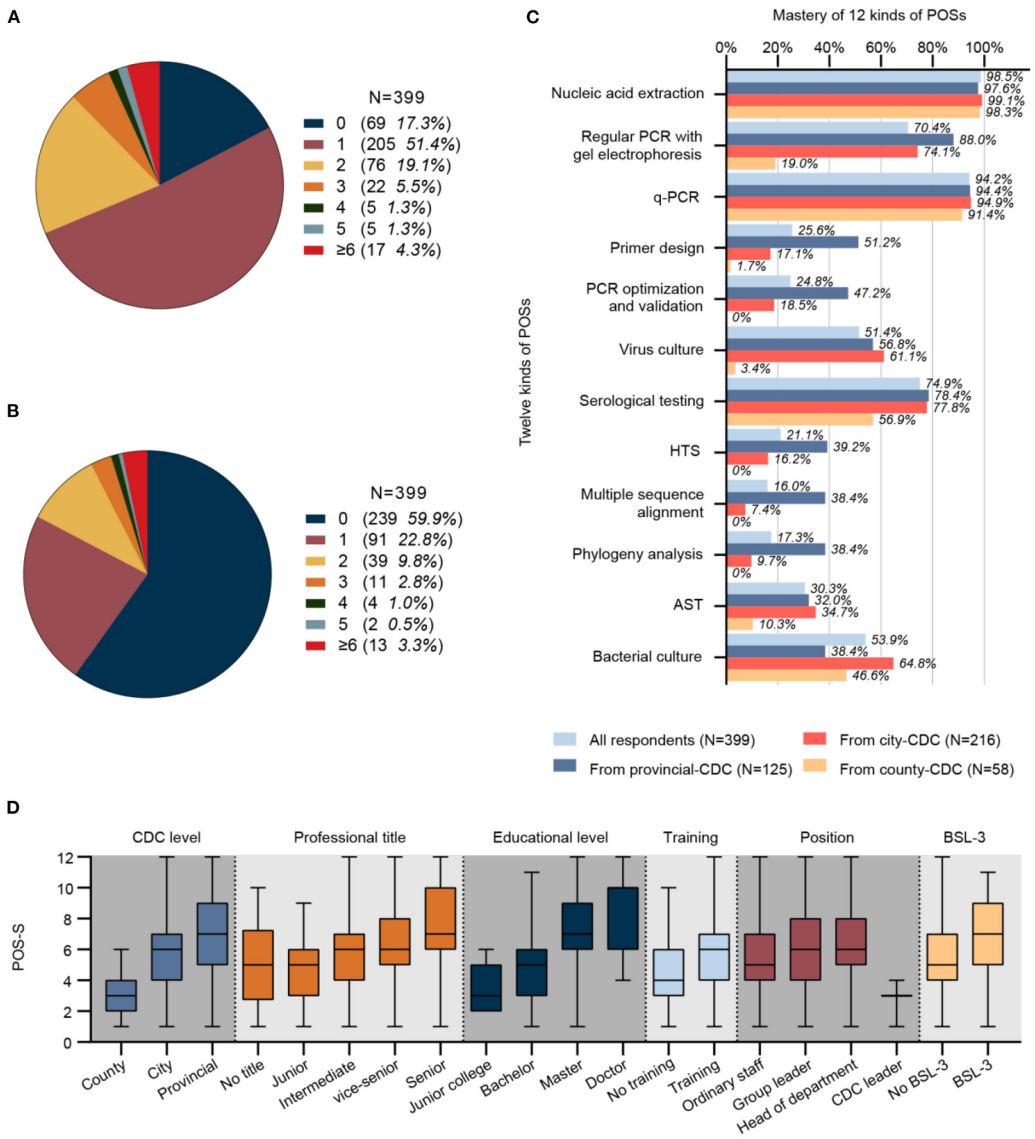
**(A)** Number and proportion of respondents receiving training with different frequencies per year (*N* = 399). **(B)** Number and proportion of respondents participating in field epidemiological surveys with different frequencies per year (*N* = 399). **(C)** Mastery of 12 kinds of POS (*N* = 399). **(D)** A significant difference in POS-S was observed by CDC level, job title, educational level, training status, position, and BSL-3 laboratory among different groups (*N* = 399, *P* values were 4.40E-21, 1.58E-7, 8.60E-21, 2.53E-5, 6.13E-4 and 7.99E-6, respectively). CDC, center for disease control and prevention; q-PCR; quantitative real-time polymerase chain reaction; AST, antimicrobial susceptibility test; HTS, high-throughput sequencing; POS-S, personal occupational skill score; BSL, biosafety level.

A total of 82.7% (330/399) of respondents had participated in theoretical or operational training related to nucleic acid detection to varying degrees every year (Figure 3A), and 40.1% (160/399) of respondents had field epidemiological survey work experience to varying degrees every year (Figure 3B).

#### Mastery of personal occupational skills (POSs)

The mastery of 12 different POSs was investigated (Figure 3C). POSs with high mastery (>80%) included nucleic acid extraction (98.5%) and q-PCR (94.2%). POSs with relatively high mastery (60–80%) included regular PCR with gel electrophoresis (70.4%) and serological testing (74.9%). POSs with intermediate mastery (40–60%) included virus culture (51.4%) and bacterial culture (53.9%). POSs with relative low mastery (20–40%) included primer design (25.6%), PCR optimization and validation (24.8%), HTS (21.1%), and AST (30.3%). POSs with low mastery (<20%) included multiple sequence alignment (16.0%) and phylogenetic analysis (17.3%).

### Detection capacity of p-labs during a pandemic and key affecting factors

#### Changes and current status of maximum detection capacity (MDC) before and after the COVID-19 pandemic

**Figure 4:**
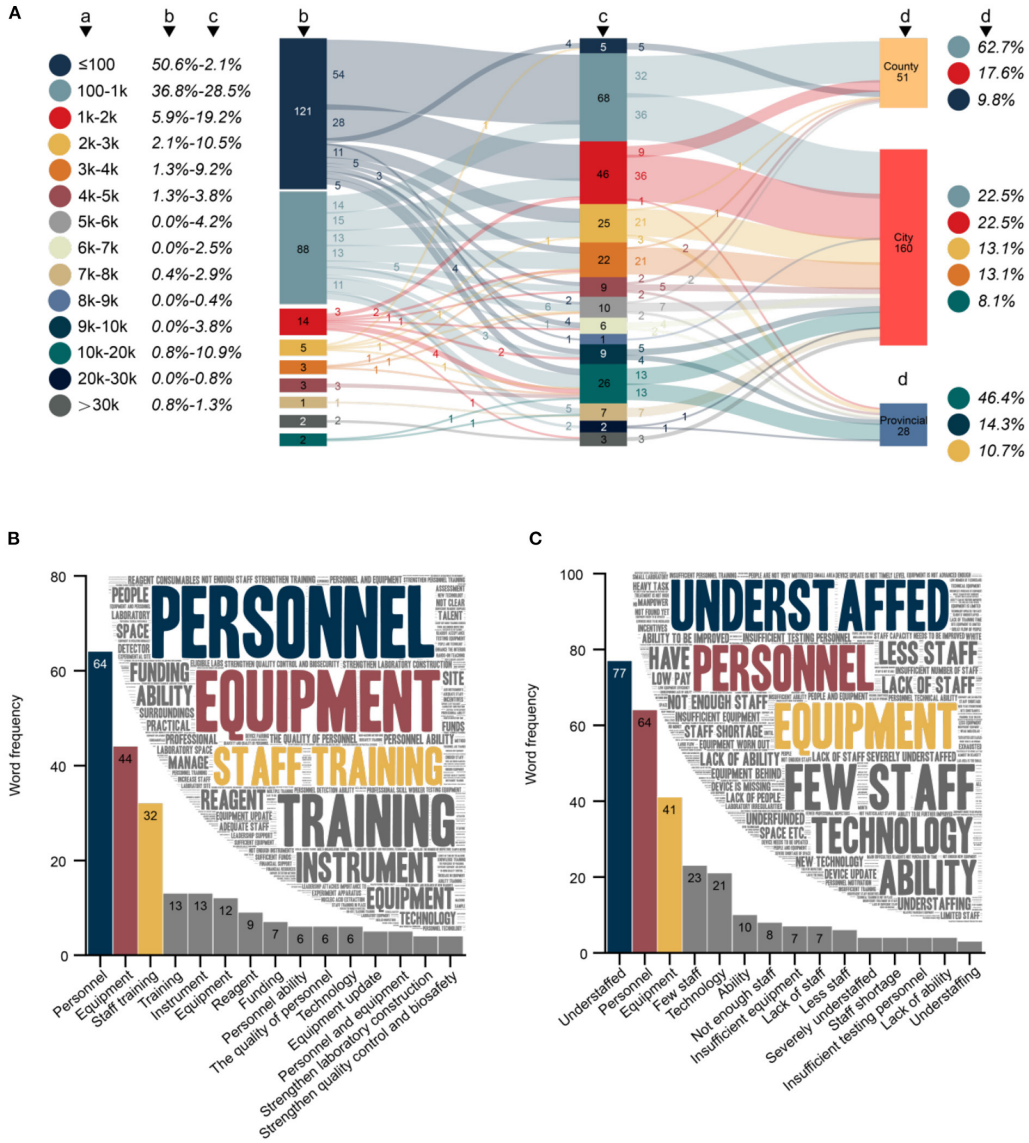
**(A)** Changes and current status of maximum detection capacity (MDC) before and after the COVID-19 outbreak (*N* = 239). (a) MDC. (b) Number and proportion of CDCs with different MDCs before the COVID-19 outbreak. (c) Number and proportion of CDCs with different MDCs after the COVID-19 outbreak. (d) Current status of MDC in different levels of CDC (only the top three most common MDCs are listed). **(B)** Word cloud and word frequency of top 15 words regarding the key to improving p-lab capacity (*N* = 386). **(C)** Word cloud and word frequency of top 15 words regarding difficulties in the p-lab (*N* = 374). Chinese synonyms may be translated into the same English vocabulary in **(B,C)**.

A total of 98.7% (394/399) respondents indicated that the CDC with which they were affiliated had established an expert working group and contingency plans in response to the COVID-19 pandemic. Before the COVID-19 outbreak, 50.6% (121/239) of CDCs in China could test no more than 100 swab samples per day (Figure 4A); this proportion has decreased to 2.1% (5/239 CDCs, all county-level CDCs). The MDC of these CDCs mainly showed exponential growth, among which 44.6% (54/121) has increased to 100–1 k level, 23.1% (28/121) has increased to 1–2 k level, and even 4.1% (5/121) has directly increased by two orders of magnitude, reaching a breakthrough in MDC of over 10 k.

The MDC of CDCs (36.8%, 88/239) that previously had the MDC of 100–1 k showed a several-fold improvement (69.3%, 61/88) after the COVID-19 outbreak. These favorable changes have prompted 62.7% (32/51) of county CDCs to reach the MDC of 100–1 k, 62.5% (100/160) of city CDCs to reach the MDC of 1-10 k, and 46.4% (13/28) of provincial CDCs to reach the MDC of more than 10 k.

#### Factors related to POS-S

The median POS-S of 339 respondents was 6, with a mean 5.8 (standard deviation 2.5). The POS-S was not normally distributed, and a significant difference was observed in the factors of CDC level, job title, educational level, position, training status, and BSL-3 laboratory among different groups (Figure 3D). The median POS-S for county, city, and provincial CDC p-lab staff was 3, 6, and 7, respectively. The median POS-S for the group with no professional title, a junior title, intermediate title, vice-senior title, and senior title was 5, 5, 6, 6, and 7, respectively. The median POS-S of respondents with junior college, bachelor's, master's, and doctorate degrees was 3, 5, 7, and 10, respectively. The median POS-S of respondents who were general p-lab staff, a group leader, department leader, and CDC leader was 5, 6, 6, and 3, respectively. The median POS-S of respondents who never had and those who had participated in theoretical or operational training was 4 and 6. The median POS-S of respondents affiliated with a CDC that did not have BSL-3 facilities and a CDC that had BSL-3 facilities was 5 and 7.

#### Factors related to IEI-S

**Figure 5:**
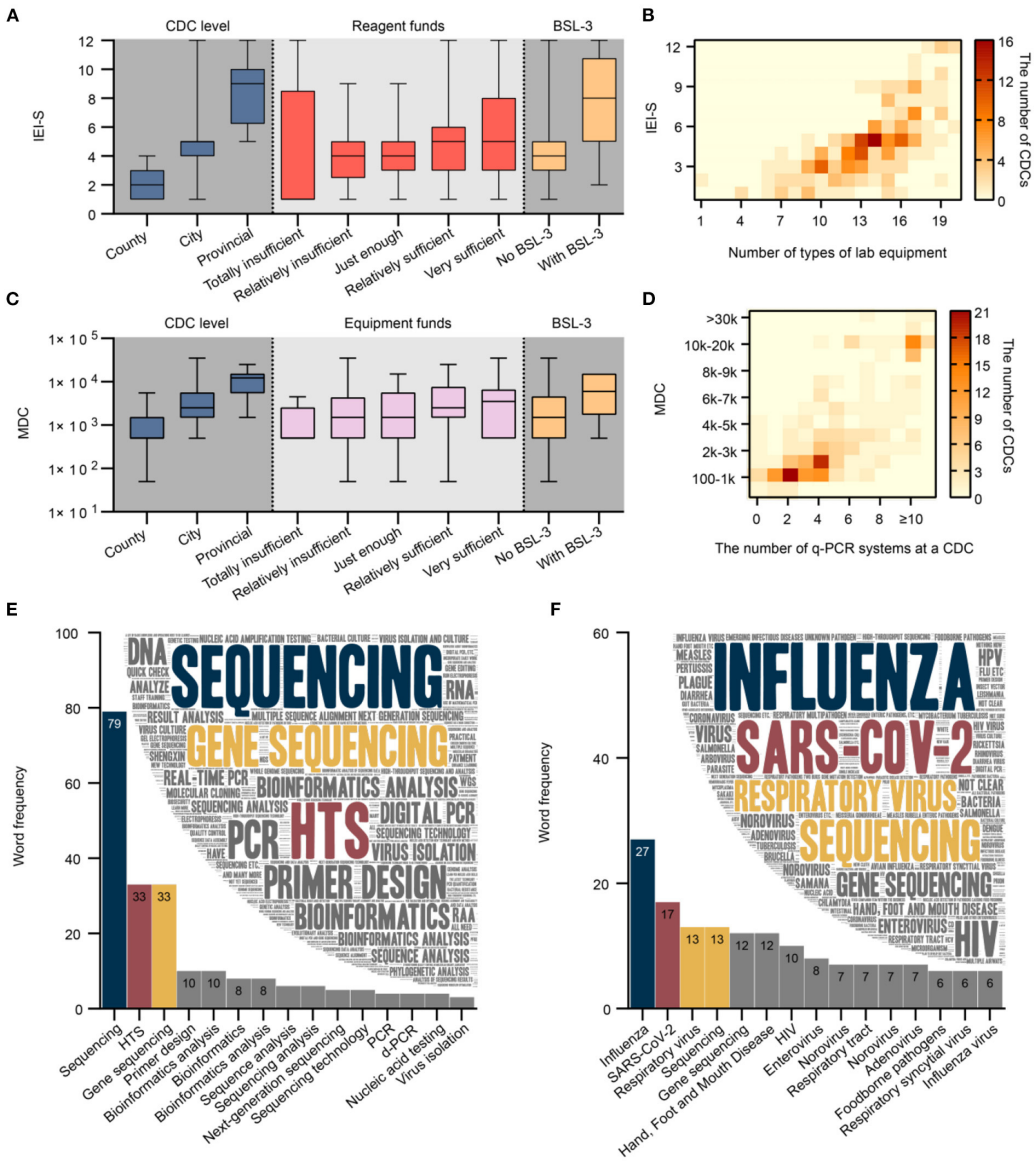
**(A)** Significant difference in IEI-S was observed by CDC level, reagent funds, and BSL-3 laboratory among different groups (*N* = 239, *P* values were 2.06E-26, 0.04 and 3.07E-7, respectively). **(B)** There was a strong and significant correlation between IEI-S and the number of laboratory equipment types (*N* = 239). **(C)** A significant difference was observed in the MDC by CDC level, equipment funds, and BSL-3 laboratory among different groups (*N* = 239, *P* values were 5.79E-18, 0.03 and 1.59E-3, respectively). **(D)** There was a strong and significant correlation between MDC and the number of q-PCR systems (*N* = 239). **(E)** Word cloud and word frequency of top 15 words regarding laboratory skills or techniques of concern to CDC staff (*N* = 380). **(F)** Word cloud and word frequency of top 15 words regarding pathogens of concern to CDC staff (*N* = 349). CDC, center for disease control and prevention; q-PCR; quantitative real-time polymerase chain reaction; MDC, maximum detection capacity; BSL, biosafety level; IEI-S, implementable experimental item score. Chinese synonyms may be translated into the same English vocabulary in **(E,F)**.

The median IEI-S among the 239 CDCs was 5, with a mean 4.7 (standard deviation 2.4). The IEI-S was not normally distributed, and a significant difference was observed in the factors of CDC level, reagent funds, and BSL-3 laboratory among the different groups (Figure 5A). The median IEI-S for county, city, and provincial CDCs was 2, 5, and 9, respectively. The median IEI-S for the group reporting reagent funds as being totally insufficient, relatively insufficient, just enough, relatively sufficient, and very sufficient was 1, 4, 4, 5, and 5, respectively. The median IEI-S for CDCs without and with a BSL-3 laboratory was 4 and 8, respectively. There was a significantly strong correlation between IEI-S and the number of laboratory equipment types (Spearman correlation coefficient = 0.725). Having 14 different types of laboratory equipment and implementing 5 kinds of IEI was the most common status of a CDC (Figure 5B); 15 and 8, 17 and 9, 19 and 12 were the most common status of the provincial CDCs; 14 and 5 was the most common status of the city CDCs; 10 and 3 was the most common status of the county CDCs.

#### Factors related to MDC

The median MDC of the 239 CDCs was 2,500. The MDC was not normally distributed, and a significant difference in CDC level, equipment funds, and BSL-3 laboratory among different groups was observed (Figure 5C). The median MDC for county, city, and provincial CDCs was 500, 2500, and 12,250, respectively. The median MDC for groups reporting equipment funds as being totally insufficient, relatively insufficient, just enough, relatively sufficient, and very sufficient was 500, 1,500, 1,500, 2,500, and 3,500, respectively. The median MDC for CDCs without and with a BSL-3 laboratory was 1,500 and 6,000, respectively. There was a significantly strong correlation between MDC and the number of q-PCR systems (Spearman correlation coefficient = 0.740). In general, the MDC was 100–1 k for CDCs with 2 q-PCR systems, 1–2 k with 4, more than 10 k with ≥10 systems (Figure 5D).

### Challenges and outlook of p-labs

#### Difficulties in improving p-lab capacity

A total 96.7% (386/399) of respondents answered the open-ended question regarding keys to improving the p-lab capacity (Figure 4B). The content of the 386 responses totaled 4,799 Chinese characters, from which 513 keywords could be extracted with a cumulative word frequency of 779. The top three keywords in word frequency were personnel (8.2%, 64/779), equipment (5.6%, 44/779), and staff training (4.1%, 32/779). A total 93.7% (374/399) of respondents answered the open-ended question regarding the difficulties faced in p-labs (Figure 4C). The content of the 374 answers totaled 3,791 Chinese characters, from which 388 keywords could be extracted with a cumulative word frequency of 678. The top three keywords in word frequency were understaffed (11.4%, 77/678), personnel (9.4%, 64/678), and equipment (6.0%, 41/678).

#### Laboratory skills and pathogens of concern to CDC staff

A total of 95.2% (380/399) of respondents answered the open-ended question, “What personal occupational skills or techniques do you currently need to learn or improve most?” (Figure 5E). A total of 268 keywords were extracted from all responses (2,903 Chinese characters) with a cumulative word frequency of 498. The top three words in word frequency were sequencing (15.9%, 79/498), HTS (6.6%, 33/498), and gene sequencing (6.6%, 33/498). A total 87.5% (349/399) of respondents answered the open-ended question, “Which pathogens do you need to test or study in the future?” (Figure 5F). A total of 312 keywords were extracted from the answers (2,721 Chinese characters) with a cumulative word frequency of 549. The top three words in word frequency were SARS-CoV-2(4.9%, 27/549), influenza (3.1%, 17/549), respiratory viruses (2.4%, 13/549), and sequencing (2.4%, 13/549).

## Discussion

Prompt and accurate feedback from the laboratory is essential for confirmation of cases and decision-making, which is the premise and foundation guiding public health responses. Monitoring and evaluating laboratory capacity with an appropriate approach and methodology play a critical role, especially in the context of the COVID-19 pandemic (2, 6, 7). Consistent with national guidance, early discovery and field controlling of infectious diseases is the core function of county-CDC while city-CDC should put more attention on MDC, IEI and biosafety. Absolute accuracy diagnosis of pathogens and rapid response to the public health emergencies was designated as the provincial-CDC's responsibility. To the best of our knowledge, this study was the first nationwide cross-sectional survey to investigate the p-lab status and capacity in CDCs of China and to compare the workload before and after the COVID-19 pandemic. Considering that public health system reform in China is attracting widespread attention owing to the valuable experiences and problems that have arisen in response to the current pandemic, the results of this study provide a basis for the Chinese government to adjust health policies in response toCOVID-19 and to help other developing countries.

Curbing the spread of the COVID-19 pandemic requires the joint efforts of individuals, government, and society as a whole, among which an administrative coordinator with executive abilities is critical and essential (8, 9). One manifestation of the government's priorities that nearly all CDCs have established expert groups and emergency plans against COVID-19, and adequate funds for reagents and equipment show that this interest has been converted into tangible emergency supplies, which is the underlying reason for the dramatic changes in work hours and detection capabilities before and after the start of the pandemic.

The increase in working hours directly reflects the burden of stress faced by CDC staff. Unfortunately, however, the rewards do not match the high-intensity workload owing to the insufficient staff funds. Working more than 8 h per day has become the norm for p-lab staff since the initial COVID-19 outbreak, which was not anticipated when designing the questionnaire, making it reasonable to infer that the actual increment in working hours is far more than 2 h. The work burden may gradually be mitigated for laboratory staff as the COVID-19 pandemic is gradually controlled worldwide, but the duration remains elusive (10, 11).

The combination of multiple diagnostic techniques, such as nucleic acid testing and antibody testing, contributes to reducing the false-negative rate inSARS-CoV-2 detection; therefore, diversification of laboratory equipment warrants attention and investment (12). Our survey suggests that the LEP of biosafety, storage, and centrifugation-related equipment at the CDCs was generally high. Autoclaves for consumable sterilization were less prevalent than those for biohazard waste, possibly because high-quality consumables such as independently packaged sterile pipette tips with filters are well-funded. A low-temperature freezer was relatively uncommon at county-level CDCs, possibly because biological samples are typically transported to city-level CDCs promptly after collection with no requirement for long-term frozen storage.

Multiple countries have been successful at controlling SARS-CoV-2 transmission by investing in large-scale testing capacity (13). A score instruments in SARS-CoV-2 detection, the BSC and q-PCR system plays a decisive role in the MDC of a CDC; hence, the precise number and allocation of these were further investigated. The p-lab of each CDC should have no <3 BSCs and 4 q-PCR systems, with the median as the reference standard, except for county-level CDC, for which the requirement should be commensurately relaxed. CDCs could have 1.3 (1,209/947 = 1.277) q-PCR systems per BSC to fully exploit the hardware resources; this ratio was optimized after years of CDC laboratory work rather than derived from stipulations in documental norms. The high LEP of an automatic extractor has prompted nucleic acid extraction no longer being the rate-limiting step in virus detection; as a result, a highly automated p-lab possesses robust SARS-CoV-2 testing capacity (14). With the progressive use of automated pipetting platforms, tube-opening and PCR plate preparation will be further simplified and standardized; accordingly, the demand for q-PCR systems will increase in a more pronounced manner than that of BSCs.

As a WHO-recommended method, q-PCR is widely used to detect SARS-CoV-2 (15). In contrast, regular PCR is currently used more often to support experiments with a scientific research purpose. Although d-PCR, known as third-generation PCR, demonstrates higher sensitivity and quantitative accuracy than q-PCR (16, 17), its low prevalence implies that absolute quantification is non-essential for SARS-CoV-2 detection and q-PCR performs well-enough. In addition to the routine screening of pathogens, the work duties of provincial-level CDCs include scientific research, method validation, and providing training and assessment to lower-level CDCs, which illustrates that the broad scope of responsibilities brings about the need for newly emerging techniques such as d-PCR.

According to the respondents, equipment is a critical factor in improving pathogen detection capacity; the statistics indicated that the diversification of laboratory equipment determines the comprehensiveness of IEIs or laboratory services. The absence of heat signal in the upper left section above the diagonal (Figure 5B) reveals interrelatedness in that strengthening of laboratory capacity lags behind investment in equipment, which indirectly indicates the indispensable nature of timely practitioner training and frequent practice. Equipment is considered the predominant difficulty presently faced by CDCs, despite being well-funded. Such a contradiction is probably due to the cumbersome procurement process of laboratory equipment at CDCs, often hindering the prompt application of advanced technology. Therefore, the relevant authorities should improve the existing procurement processes to be simpler, more efficient, and sound within the legal and compliance framework. It is consistent with common sense that staff at higher CDC levels or with higher professional titles, education, and positions have more substantial professional competency. Professional title and position are comprehensive indicators that integrate various factors, including job tenure and skill level. The findings indicated that both academic education and short-term training help increase the experimental competencies of p-lab staff, who also subjectively believe that personnel and staff training are crucial determinants to improving p-lab capacity. Given the above, the relevant authorities should develop implementable training programs and provide more learning opportunities for CDC p-lab staff, such as full-time education at university level or refresher training at a higher-level CDC (18). Staff shortages represent a grievous issue at the CDCs, which could be reasonably interpreted as a severe deficiency of staff funding with convincing evidence pointing to the directions of reforms for the introduction, motivation, and retention of laboratory personnel and technical talent.

Sufficient reagents and consumables motivate staff to repeatedly optimize experimental conditions and gain experience from failures, ultimately prompting the transformation of new technologies into stable IEIs or services, such that more the abundant the reagent funds, the higher the IEI-S of the p-lab. Although the IEI-S is associated with equipment diversity, no significant correlation was observed between IEI-S and equipment funds, suggesting that the primary way to obtain equipment was not restricted to procurement. For example, reagent suppliers are willing to provide their larger customers with accessible trial equipment for public health emergencies, such as the COVID-19 pandemic. Following the requirements of normalized prevention and control regulations enacted by the Chinese government, CDC leaders tend to prioritize spending of equipment funds on MDC by procuring BSCs and q-PCR systems; hence, the MDC is more related to equipment than reagent funds.

The vast majority of upper-level CDCs showed significant advantages in capability over lower ones, but some basic skills are not the case owing to institutional positioning and division of labor. For instance, the pathogen culture and AST ability of city-level CDCs and nucleic acid extraction ability of county-level CDCs appeared stronger than those of provincial-level CDCs. Another interesting phenomenon is that most aspects of CDCs with a BSL-3 laboratory are superior to none. Given various confounders such as CDC level, having a BSL-3 laboratory cannot be considered a facilitating factor of CDC capability but probably could be intuitively regarded as an indicator. CDCs can more easily access policy, personnel, equipment, and financial support in the process of planning, constructing, and maintaining a BSL-3 laboratory. Correspondingly, the operation of a BSL-3 laboratory would further expand the scope of services as a kind of feedback, improving the professionalism and proficiency of the p-lab team in practice. Therefore, the planning and construction of a BSL-3 laboratory could be considered the starting point for CDC p-lab improvement.

Much concern and interest about technology has been focused on the sequencing field and was frequently mentioned in open-ended questions concerning pathogens. WGS can be used to study the transmission and evolution of SARS-CoV-2, and it is increasingly recognized as a critical tool for public health responses to the COVID-19 pandemic (19). Thus, the national guidelines have placed requirements on WGS for SARS-CoV-2 at provincial CDCs, making it more prevalent than influenza WGS, which has been implemented for years. Although few p-lab staff has currently mastered the HTS, its development in the future will give rise to abundant HTS-related products and commercialized services (20, 21). In stark contrast to the pursuit of new techniques, the mastery of traditional microbiological or molecular methods, such as pathogen culture and regular PCR becomes less of a priority, especially in provincial-level CDCs.

Respiratory viruses like influenza and SARS-CoV-2 are among the most concerning pathogens but are not as intensely focused on as sequencing, which implies that monitoring and research of other infectious diseases such as hand, foot, and mouth disease or AIDS remain non-negligible in CDCs. Taking SARS-CoV-2 as an example of pathogen research, a robust testing approach against this pathogen with pandemic potential should be pre-established and include a complete repertoire of the most advanced technologies.

There are some deficiencies in this study that should be improved in future. Many county-level CDCs in China have not established a p-lab or similar department and lack interaction with the national CDC in routine work, making it challenging to include respondents from county CDCs in the survey nationwide. Although two representative provinces were carefully selected, the sample size and representativeness of county-level CDCs remain inferior to that of provincial and city CDCs. Another point worth noting is that continuous variables, such as working hours, were collected using multiple-choice questions rather than fill-in-the-blank questions with the aim to improve the convenience and efficiency of completing the questionnaire but which could reduce accuracy of the data.

The thousands of CDCs in China constitute one of the largest public health systems in the world, providing public health services to approximately one-sixth of the global population. In the context of the COVID-19 pandemic, the p-labs of China's CDCs as a whole provide vital support in the prevention and control of domestic outbreaks and contribute to curbing the global spread of infectious diseases. Although p-lab development at CDCs is unbalanced, substantial progress has been made during the COVID-19 pandemic particularly in detection capacity. As long as CDCs in China's provinces, cities, and counties strengthen and maintain a smooth communication mechanism, a strong network can be formed to cope with future public health emergencies. Human resources and sustainable funding are key to the future development of CDCs and require the attention of governments at all levels. To provide nationwide evidence, we must continue to monitor the status and evaluate the capacity of CDC p-labs in the coming years. We also suggest that the p-labs performing SARS-CoV-2 testing could improve their quality and competence according to our survey results, and regular accreditation and participation in external quality assessment are strongly recommended in the future. The upcoming reform of the public health system in China is the driving force for CDC development, and the sustainable development of CDCs in the future is expected through enhanced cooperation and interdisciplinary integration of various departments.

## Data availability statement

The original contributions presented in the study are included in the article/supplementary material, further inquiries can be directed to the corresponding author.

## Author contributions

XM and JW designed the study. PN, RZ, JL, and MN analyzed and interpreted the data. JW wrote the paper. All authors provided a critical review and approved the final manuscript.

## Conflict of interest

The authors declare that the research was conducted in the absence of any commercial or financial relationships that could be construed as a potential conflict of interest.

## Publisher's note

All claims expressed in this article are solely those of the authors and do not necessarily represent those of their affiliated organizations, or those of the publisher, the editors and the reviewers. Any product that may be evaluated in this article, or claim that may be made by its manufacturer, is not guaranteed or endorsed by the publisher.

## Footnotes

^1^ https://www.who.int/publications/i/item/9789241580410
